# Effect of cataract surgery on retinal nerve fiber layer thickness parameters using scanning laser polarimetry (GDxVCC)

**DOI:** 10.4103/0301-4738.67048

**Published:** 2010

**Authors:** Tanuj Dada, Geeta Behera, Anand Agarwal, Sanjeev Kumar, Ramanjit Sihota, Anita Panda

**Affiliations:** Glaucoma Research Facility, Dr. Rajendra Prasad Center for Ophthalmic Sciences, All India Institute of Medical Sciences, New Delhi, India

**Keywords:** Birefringence, cataract, glaucoma, scanning laser polarimeter

## Abstract

**Purpose:**

To study the effect of cataract extraction on the retinal nerve fiber layer (RNFL) thickness, and assessment by scanning laser polarimetry (SLP), with variable corneal compensation (GDx VCC), at the glaucoma service of a tertiary care center in North India.

**Materials and Methods:**

Thirty-two eyes of 32 subjects were enrolled in the study. The subjects underwent RNFL analysis by SLP (GDx VCC) before undergoing phacoemulsification cataract extraction with intraocular lens (IOL) implantation (Acrysof SA 60 AT) four weeks following cataract surgery. The RNFL thickness parameters evaluated both before and after surgery included temporal, superior, nasal, inferior, temporal (TSNIT) average, superior average, inferior average, and nerve fiber index (NFI).

**Results:**

The mean age of subjects was 57.6 ± 11.7 years (18 males, 14 females). Mean TSNIT average thickness (μm) pre- and post-cataract surgery was 49.2 ± 14.1 and 56.5 ± 7.6 (*P* = 0.001). There was a statistically significant increase in RNFL thickness parameters (TSNIT average, superior average, and inferior average) and decrease in NFI post-cataract surgery as compared to the baseline values. Mean NFI pre- and post-cataract surgery was 41.3 ± 15.3 and 21.6 ± 11.8 (*P* = 0.001).

**Conclusions:**

Measurement of RNFL thickness parameters by scanning laser polarimetry is significantly altered following cataract surgery. Post the cataract surgery, a new baseline needs to be established for assessing the longitudinal follow-up of a glaucoma patient. The presence of cataract may lead to an underestimation of the RNFL thickness, and this should be taken into account when analyzing progression in a glaucoma patient.

Scanning laser polarimetry (SLP) is an imaging technique that quantifies the retinal nerve fiber layer (RNFL) thickness. The changes in the RNFL influence the visual field; however, the damage to RNFL precedes the changes in the visual field by a number of years. The RNFL is a highly ordered array of parallel axon bundles that contain microtubules, which contribute to the birefringence.[1,2]

SLP with a variable corneal compensator (GDxVCC) provides an accurate and reproducible measurement of the RNFL thickness, based on the alteration (retardation) it produces in the polarization of light passing through it. It is thought that naturally polarizing birefringent neuronal microtubules produces retardation that is proportional to the RNFL thickness.[2,3] There are various factors that influence the measurement of RNFL thickness using SLP. The influence of other birefringent structures can lead to an erroneous measurement of the RNFL. Among the anterior segment structures, the cornea[4,5] and lens[6] also contribute to birefringence. GDx VCC helps in eliminating the anterior segment birefringence and in providing a near-accurate reading of the RNFL. However, the presence of cataract changes the birefringence produced by the lens, which cannot be compensated for by using the VCC and influences the measurement of the RNFL. Previous studies have shown the effects of cataract extraction on the measurement of RNFL, but the results have been conflicting.[7–12] The purpose of the study is to evaluate the effect of cataract surgery on RNFL thickness parameters using SLP, with individualized GDx VCC, at the glaucoma service of a tertiary eye care center in North India.

## Materials and Methods

In this prospective case series, 32 eyes of 32 subjects with age-related nuclear cataract and having IOP < 21 mmHg, normal disc, and best corrected visual acuity of 20/200 or better were recruited from the glaucoma services of a tertiary eye care center. Patients with family history of glaucoma, previous steroid use, history of ocular trauma, high myopia, with peripapillary atrophy, any other ocular disease affecting the retina or nerve fiber layer, and diabetes mellitus were excluded.

The RNFL thickness was analyzed using a commercially available GDx VCC, (Carl Zeiss Meditec, Dublin, CA, software version 5.5.1). All examinations were carried out by a single operator. The measurements were taken in an ambient light without pupillary dilatation. Three scans were obtained for each eye. The scan with the best resolution and having clearly demarcated retinal vessels was selected as the final image. Only images with a quality score of > 6 were included. Due to the influence of the corneal polarization axis and the magnitude that affects SLP measurements, making them different in all eyes, the GDx VCC employs a variable corneal polarization compensator that allows eye-specific compensation of the anterior chamber birefringence. After determining the axis and magnitude of corneal polarization in each eye by macular scanning, appropriately compensated retinal polarization images per eye were automatically obtained and combined to form one mean image used for analysis.

Based on the retardation values the software calculated the following four parameters, the TSNIT (temporal, superior, nasal, inferior, temporal) global average (average retardation under the automatically defined 3.2-mm-diameter calculation circle), superior average (average retardation beneath the measurement ellipse in the superior sector), inferior average (average retardation beneath the measurement ellipse in the inferior sector), and nerve fiber indicator (NFI) (support vector-machine-derived algorithm trained to discriminate between healthy and glaucomatous eyes).

Thirty-two eyes of 32 subjects with age-related nuclear cataract (LOCS Grade II or III) were operated upon. We chose patients with nuclear cataracts, as those with posterior subcapsular opacities did not yield useful preoperative measurements on the SLP, due to poor image quality. Only one eye from each subject was taken for the study. All eyes underwent an uneventful standard temporal, 2.75 mm, clear corneal phacoemulsification with an in-the-bag implantation of a monofocal, single piece hydrophobic acrylic intraocular lens (IOL) (Acrysof SA 60 AT, Alcon Laboratories, Fort Worth, Texas, USA). All surgeries were performed by a single surgeon (TD) under topical anesthesia. We ensured that none of the patients had any media opacities such as a posterior capsular plaque or opacification. The intraocular pressure (IOP) was ≤ 21 mmHg in all eyes.

RNFL thickness parameters were reassessed four weeks after cataract surgery in these patients.

The data of measurements pre- and post-cataract surgery were analyzed and compared using the paired t test. A *P* value of less than 0.05 was considered statistically significant.

## Results

All the eyes were taken up for postoperative measurement after four weeks. The preoperative visual acuity ranged from 20/60 to 20/200. The postoperative visual acuity of all patients was 20/20.

**Figure 1:**
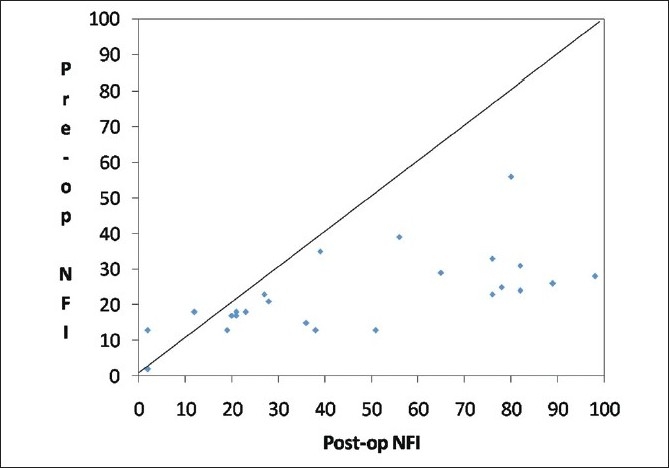
Scatter plot of pre-surgery vs. post-surgery nerve fiber index (NFI)

Mean patient age was 57.6 ± 11.7 years with a range of 38 to 73 years. There were 18 males and 14 females who qualified for the study. Mean TSNIT average thickness (μm) pre- and post-cataract surgery was significantly higher postoperatively (preoperatively 49.2 ± 14.1 and postoperatively 56.5 ± 7.6,*P* = 0.001). The mean NFI also improved significantly on postoperative measurement (preoperatively 41.3 ± 15.3 and postoperatively 21.6 ± 11.8,P = *P* = 0.001). The difference was statistically significant (*P* < 0.001) even when the non-parametric test (Wilcoxon signed rank test) was used to compare the pre-NFI and post-NFI values. Fig. 1 shows the decrease in NFI values post surgery. The other values such as superior average and inferior average also showed statistically significant improvement [Table 1].

**Table 1:** Results of scanning laser polarimetry (GDx VCC) before and after cataract surgery

RNFL Parameters	Preoperative	Post operative	% change	*P* value
TSNIT average	49.2 ± 14.1	56.5 ± 7.6	14.83	0.001
Superior average	51.6 ± 12.2	59.8 ± 7.3	15.89	0.004
Inferior average	50.2 ± 13.7	61.5 ± 10.3	22.51	0.001
NFI	41.3 ± 15.3	21.6 ± 11.8	47.69	0.001

RNFL: Retinal nerve fiber layer, NFI: Nerve fiber index

**Figure 2:**
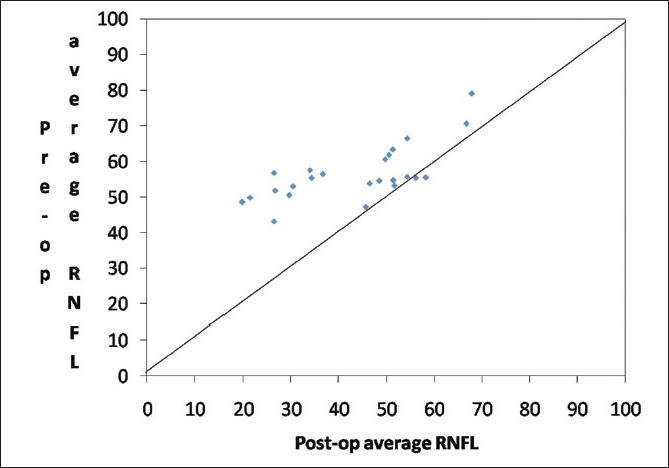
Scatter plot of pre-surgery average RNFL vs. post-surgery average retinal nerve fiber layer thickness (RNFL)

**Figure 3:**
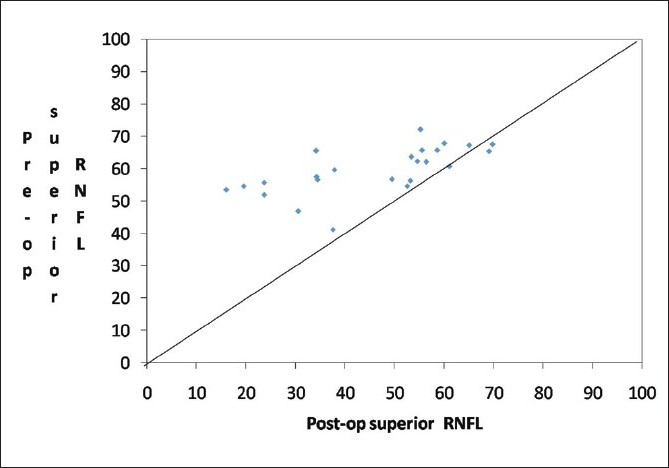
Scatter plot of pre-surgery superior RNFL vs. post-surgery superior retinal nerve fiber layer thickness (RNFL)

**Figure 4:**
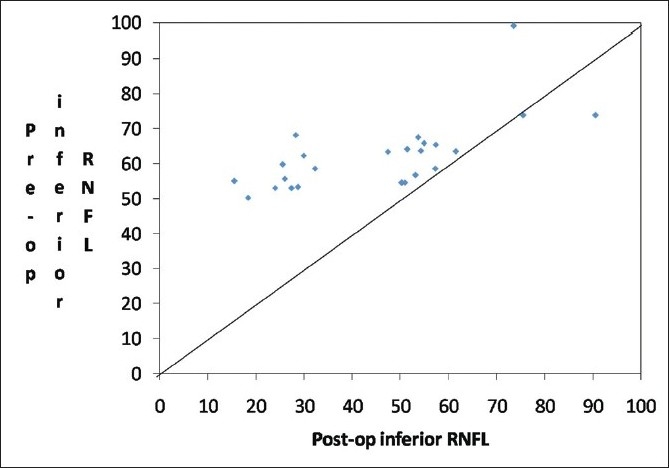
Scatter plot of pre-surgery inferior RNFL vs. post-surgery inferior retinal nerve fiber layer thickness (RNFL)

**Figure 5:**
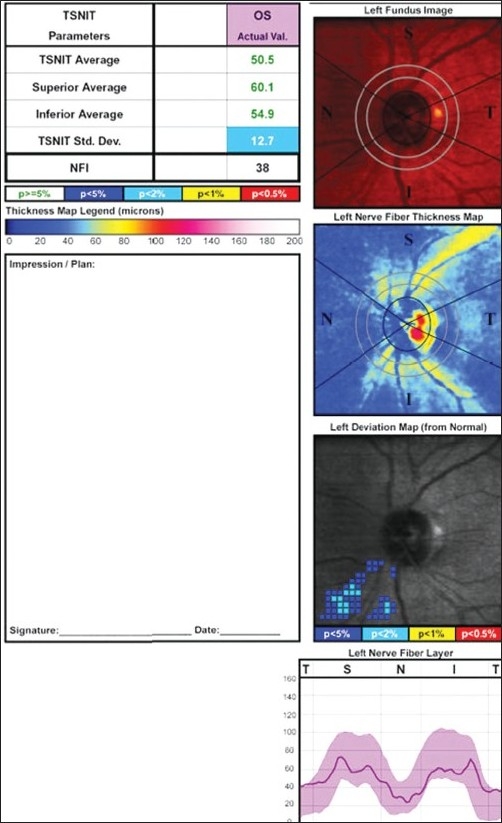
Scanning Laser Polarimetry printout of a patient with nuclear sclerosis

**Figure 6:**
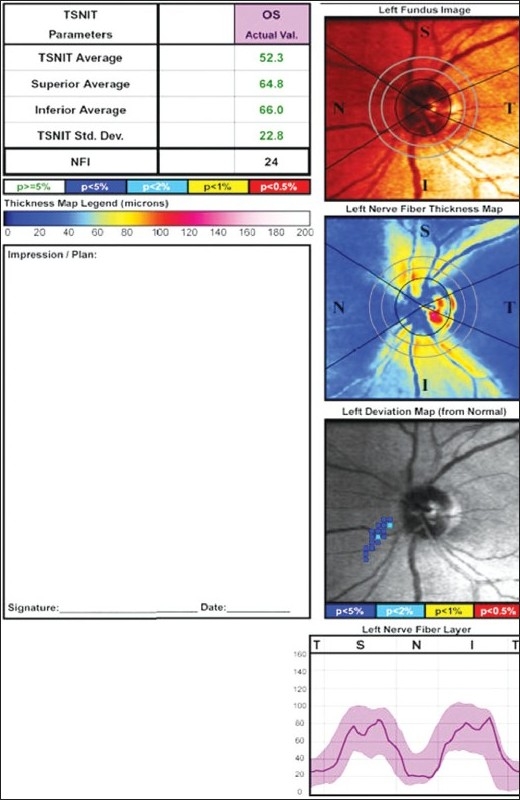
Scanning Laser Polarimetry output of the same patient showing improved values postoperatively

There was a significant increase in the RNFL thickness parameters (TSNIT average, superior average, and inferior average) and decrease in NFI post cataract surgery as compared to the baseline values in the eyes implanted with hydrophobic acrylic IOLs [Figs 2,3 and 4]. Figs 5 and 6 are preoperative and postoperative SLP printout of a patient with nuclear sclerosis respectively.

## Discussion

Scanning laser polarimetry employs the principle of birefringence of the RNFL. Unfortunately, birefringence is also a property of the cornea and to a small extent of the lens. The GDx VCC of the current version of SLP excludes the effect of the cornea on the measurements, but that of the cataractous lens, albeit small, remains to be determined. In a preliminary laboratory investigation, Kremmer *et al*,[8] did not find any changes in the polarized light when it was passed through the IOLs of different materials, but their study did not include Acrysof SA 60 AT, Alcon, which was the IOL we used in our study.

Other investigators have shown the influence of cataract on the RNFL parameters, on SLP.[7–14] However; their results have been very conflicting and are confounded by the use of different IOL types.

Gazzard *et al*.,[7] in a similar study on glaucoma patients, have shown a significant change in SLP measurements after cataract extraction, especially so in patients with posterior subcapsular cataract. They recommended the establishment of a new baseline in glaucoma patients following cataract surgery. Kremmer *et al*.[8] have also concluded that after cataract surgery with IOL implantation, some SLP values are altered significantly, whereas, scanning laser tomography parameters are not influenced. These findings are of clinical interest, especially in the follow-up of glaucoma patients.

Park *et al*,[11] in their study on the effect of cataract extraction with different IOLs have found a statistically significant change in the mean SLP values in eyes that received an acrylic IOL (Acrysof SA 60 AT, Alcon). They postulated that the presence of ‘glistenings,’ attributed to intralenticular water vacuoles, which are a peculiarity of the Acrysof IOL, may have contributed to the effect on the SLP, but this merits further investigation. They also noted clinically important changes (15% or greater) in SLP measurements irrespective of the IOL type in individual cases. Hence, it is probable that the change observed is due to the removal of the cataractous lens, the effect of which is heretofore undetermined.

Contrary to all the reports mentioned above, Vetrugno *et al*,[12] have shown no statistically significant differences between SLP parameters before and after cataract surgery, regardless of the type of IOL implanted.

Our study has concluded that there is a definite influence of cataractous lens removal on all the parameters of RNFL measurement using SLP with GDx VCC. The presence of nuclear sclerosis and cataract leads to an underestimation of the various RNFL parameters, due to the retardation, probably caused by increased birefringence of the cataractous lens. This is found to be statistically significant with each GDx VCC parameter.

The effect of posterior capsular opacification (PCO) on SLP parameters appears to be the opposite of the effect of the presence of cataract, as has been shown by Garcia Medina *et al*.[15] In their study, PCO leads to an overestimation of RNFL thickness, which is taken care of once the PCO is tackled with Nd : YAG Laser capsulotomy.

Garcia Medina *et al*,[16] studied the effect of PCO on SLP parameters and the effect of its removal on 26 eyes of 26 patients, who developed PCO following cataract surgery. They concluded that GDx VCC measurements, especially those related to the corneal polarization axis and magnitude, were significantly altered following Nd : YAG laser capsulotomy and so were the GDx VCC parameters.

In a patient with glaucoma, the lens may develop cataractous changes as a consequence of age, which is accelerated after glaucoma filtering surgery as also with the use of various anti-glaucoma medications. The presence of cataract causes an apparent decrease in the RNFL measurement parameters, which makes the assessment of glaucoma in these patients fallacious. This apparent decrease in the RNFL thickness may be mistaken as progression. Consequently, the patient may be erroneously subjected to additional medical therapy or glaucoma filtering surgery. After cataract surgery, the increase noted in the RNFL thickness parameters of the SLP and the decrease noted in the NFI is just an apparent change rather than a true change. For longitudinal evaluation of glaucomatous eyes that develop a cataract, a possible additional lens effect on the anterior segment birefringence and its effect on the measurement of RNFL thickness should be considered.

This can be explained by the possibility of retardation caused by the cataract or it may be due to the influence of the IOL material. However, the former seems to be a more probable cause for this apparent change.

Our study was limited by the lack of RNFL parameters on the GDx VCC before the onset of cataract, to prove the same. Our protocol involved the use of a 2.75 mm, clear corneal incision that induced very little change in astigmatism.[17] Therefore, any slight change in astigmatism affecting the SLP result remained undetermined as the anterior segment birefringence correction was assessed only before cataract surgery,

The NFI, which was obtained by using the neural network technique, was a unique parameter that helped in the determination and quantification of the glaucomatous damage as mild, moderate or advanced. The NFI was described as a very sensitive index of glaucomatous damage with the highest area under the curve (AUC), in a few studies.[18–20] In our study the greatest change was noted in the NFI, postoperatively. For patients with documented advanced or moderate glaucoma, post cataract surgery, the apparent decrease in the NFI value would give a false indication of clinical improvement.

As a corollary, in any patient on long-term follow-up of glaucoma on the GDx VCC, who shows evidence of progression, lenticular evaluation for development of nuclear sclerosis becomes important. This is also an important consideration for ocular hypertensives who develop cataract during the course of their follow-up and are being assessed for progression to glaucoma using GDx VCC.

We recommend that following cataract surgery, establishing a new baseline would be most appropriate for monitoring all glaucoma patients on the GDx VCC.
